# Structural basis for the selective methylation of 5-carboxymethoxyuridine in tRNA modification

**DOI:** 10.1093/nar/gkad668

**Published:** 2023-08-17

**Authors:** Jaehun Yoo, Jangmin Lee, Jungwook Kim

**Affiliations:** Department of Chemistry, Gwangju Institute of Science and Technology, Gwangju 61005, Korea; Department of Chemistry, Gwangju Institute of Science and Technology, Gwangju 61005, Korea; Department of Chemistry, Gwangju Institute of Science and Technology, Gwangju 61005, Korea

## Abstract

Posttranscriptional modifications of tRNA are widely conserved in all domains of life. Especially, those occurring within the anticodon often modulate translational efficiency. Derivatives of 5-hydroxyuridine are specifically found in bacterial tRNA, where 5-methoxyuridine and 5-carboxymethoxyuridine are the major species in Gram-positive and Gram-negative bacteria, respectively. In certain tRNA species, 5-carboxymethoxyuridine can be further methylated by CmoM to form the methyl ester. In this report, we present the X-ray crystal structure of *Escherichia coli* CmoM complexed with tRNA^Ser1^, which contains 5-carboxymethoxyuridine at the 5′-end of anticodon (the 34^th^ position of tRNA). The 2.22 Å resolution structure of the enzyme-tRNA complex reveals that both the protein and tRNA undergo local conformational changes around the binding interface. Especially, the hypomodified uracil base is flipped out from the canonical stacked conformation enabling the specific molecular interactions with the enzyme. Moreover, the structure illustrates that the enzyme senses exclusively the anticodon arm region of the substrate tRNA and examines the presence of key determinants, 5-carboxymethoxyuridine at position 34 and guanosine at position 35, offering molecular basis for the discriminatory mechanism against non-cognate tRNAs.

## Graphical Abstract

**Graphical Abstract:**
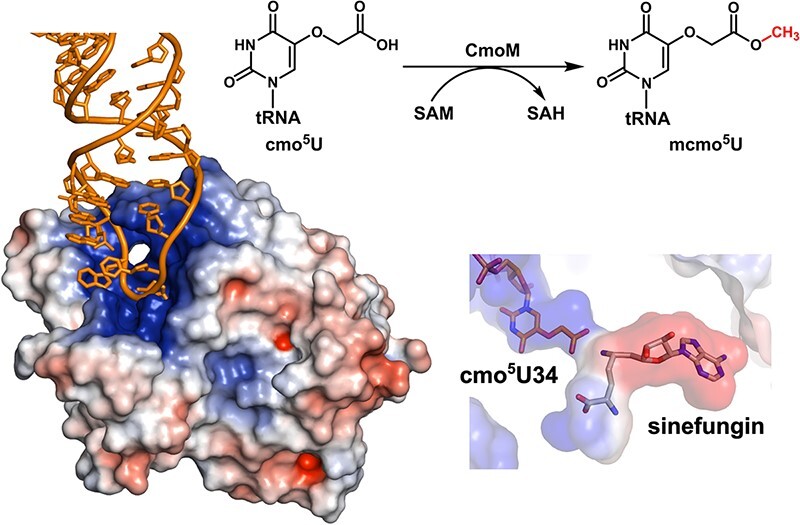

## INTRODUCTION

Transfer RNA (tRNA) delivers a codon-specific amino acid to ribosome playing a central role as an adaptor during translation (1). Most RNA molecules undergo posttranscriptional modifications; over 150 distinct modifications have been identified in RNA, where 80% of those are found in tRNAs (2). On average, bacterial and eukaryotic tRNA contains 8 and 13 modifications per molecule (3), respectively. Lack of proper post-transcriptional modifications in tRNA affects mRNA decoding capabilities (4), cellular growth (5) and regulatory function (6). In humans, certain irregular tRNA modifications have been associated with neurodegenerative and metabolic diseases (7).

**Figure 1.**
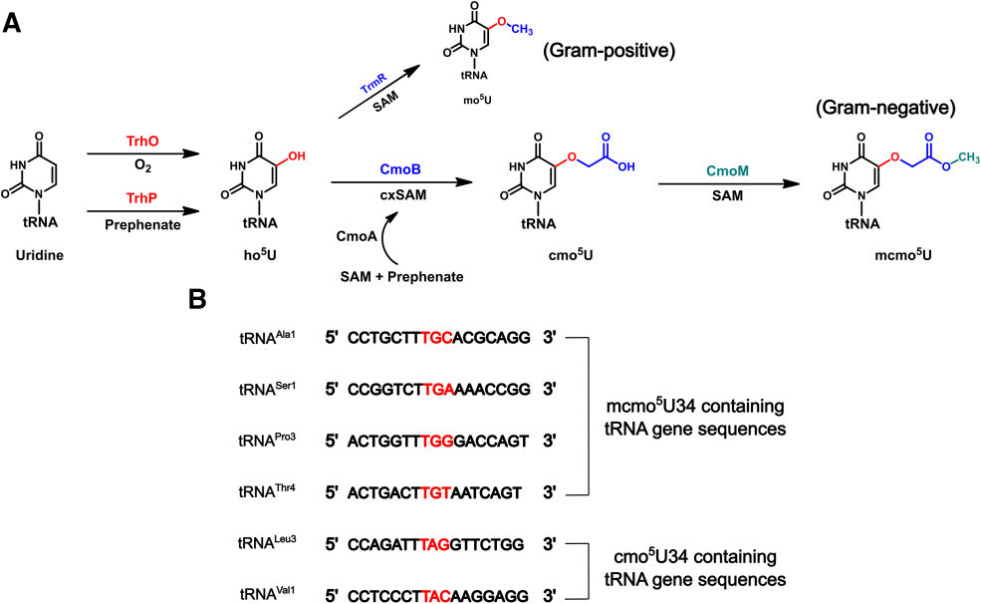
xo-type wobble uridine modifications in bacterial tRNA. (**A**) Summary of the biosynthesis of xo^5^U34 modification in bacterial tRNA. Colors in chemical structures match those of the enzymatic activity. (**B**) Gene sequences that encode tRNA anticodon arm containing cmo^5^U34 and mcmo^5^U34. Anticodon is colored in red.

Uridines at the wobble position (position 34) are almost invariably modified in cytosolic tRNA (8,9), especially at the C2 and C5 atoms of the uracil nucleobases (10). In bacteria, wobble uridine modifications fall into two groups, 5-methyluridine (xm^5^U) or 5-hydroxyuridine (xo^5^U) derivatives (11). A number of xo^5^U-type modifications have been found in both Gram-negative and Gram-positive bacteria, including 5-carboxymethoxyuridine (cmo^5^U) and 5-methoxycarbonylmethoxyuridine (mcmo^5^U) for the former, and 5-methoxyuridine (mo^5^U) for the latter. Lately, biosynthetic pathways for xo^5^U-type modifications have been fully characterized as summarized in Figure 1A. In the first step, U34 is hydroxylated at C5 to yield 5-hydroxyuridine (ho^5^U) by O_2_-dependent TrhO or prephenate-dependent TrhP (12,13). In Gram-positive bacteria, TrmR converts ho^5^U to mo^5^U using *S*-adenosyl-l-methionine (SAM) as the methyl donor (14). In Gram-negative bacteria, CmoB converts ho^5^U to cmo^5^U employing a unique metabolite carboxy-SAM (cxSAM) as the carboxymethyl donor, which is assembled from prephenate and SAM by CmoA (15,16). The wobble cmo^5^U in certain tRNAs are further methylated by SAM-dependent CmoM to produce its methyl ester, mcmo^5^U. Additionally, 2′-O-ribose methylation of mcmo^5^U occurs occasionally (∼4.2%) by TrmL to yield mcmo^5^Um in tRNA^Ser1^ (17).

The tRNA-modifying activity and the specificity of *E. coli* CmoM was first reported by Suzuki and his colleagues, who identified that mcmo^5^U34 was present in tRNA^Ala1^, tRNA^Ser1^, tRNA^Pro3^ and tRNA^Thr4^, whereas cmo^5^U34 was in tRNA^Leu3^ and tRNA^Val1^ (17) (Figure 1B). Authors also demonstrated that the terminal methyl group of the mcmo^5^U contributed to preventing frameshift during decoding GCG codon, although no detectable growth defects could be observed with *cmoM*-deficient mutant cells. CmoM belongs to Class I SAM-dependent methyltransferase family and the SAM-bound structure of CmoM from *E. coli str. O157:H7* is available (PDB ID: 4HTF). However, it is not clear how the enzyme interacts with a specific tRNA substrate and installs the methyl group on the hypomodified wobble uridine as this structure lacks RNA component.

Here, we report an X-ray crystal structure of *E. coli* CmoM complexed with sinefungin, an analog of SAM, and cellularly expressed *E. coli* tRNA^Ser1^ containing cmo^5^U at the wobble position. The 2.22 Å resolution complex structure clearly unveils detailed molecular interactions between cmo^5^U and the enzyme at atomic level, providing critical clues to the molecular basis for discriminating the cognate versus non-cognate tRNAs. Combined with the structural information, we further verified the specificity elements within both tRNA and the enzyme by examining the cellular and *in vitro* activities of CmoM variants.

## MATERIALS AND METHODS

### Cloning and protein purification of CmoM variants

*E. coli cmoM* gene implemented on pCA24N vector was obtained from the *E. coli* ASKA library (NBRP, Japan). Point mutations were introduced by *in vivo* mutagenesis as described previously (18). Primers used for cloning are listed in Supplementary Table S1. Sequences were verified by standard sequencing (Macrogen, Korea). *E. coli* BL21 (DE3) cells were transformed with the vectors harboring a *cmoM* variant, grown in LB containing 100 μg/ml ampicillin at 37°C, and induced with 0.5 mM Isopropyl β-D-1-thiogalactopyranoside (IPTG) at an OD_600_ of ∼0.6. Cells were further incubated at 20°C overnight and harvested by centrifugation. Cell pellets were resuspended in lysis buffer (25 mM HEPES (pH 7.5) and 150 mM NaCl) and sonicated for 30 min. The lysate was centrifuged at 39 000 *g* for 30 min and the supernatants were applied to the HisTrap HP column (GE Healthcare) charged with Ni^2+^. The column was equilibrated with wash buffer (25 mM HEPES (pH 7.5), 300 mM NaCl, and 20 mM imidazole) and eluted with a linear gradient of imidazole (0.02–0.5 M). Purified protein was analyzed by SDS-PAGE and buffer exchanged with the lysis buffer using Amicon centrifugal filters (Merck). An extinction coefficient of ϵ_280_ = 42 400 M^−1^ cm^−1^ was used to calculate the concentration of the protein.

### Determination of molecular weight of CmoM by size exclusion chromatography

The molecular weight of recombinant CmoM was determined by size exclusion using Superdex 75gl column (GE Healthcare, Life Sciences) on NGC FPLC system (Biorad). 100 μg of the wild-type CmoM (calculated monomer molecular weight = 31.7 kDa) was loaded onto the column with mobile phase (25 mM HEPES (pH 7.5) and 150 mM NaCl) at a flow rate of 0.5 ml/min. Elution of ovalbumin (44 kDa) and MnmC (76 kDa) from the column was also tested under the identical condition for comparison.

### Purification of *in vivo* transcribed tRNA

The plasmid pBSTNAV (Addgene, USA) was used as the tRNA expression vector which contains LPP promoter and rrnC terminator. *E. coli str. K-12 substr. MG1655 serT* (tRNA^Ser1^(TGA)) gene was amplified by polymerase chain reaction from synthetic DNA template (IDT, USA). The primers used are summarized in Supplementary Table S1. The pBSTNAV vector and the amplified PCR fragments were double digested in 37°C water bath for 2 h using the restriction enzymes EcoRI (Enzynomics, Korea) and HindIII (Enzynomics, Korea). Digested products were purified by agarose gel electrophoresis. Next, ligation step was performed using T4 ligase (Enzynomics, Korea) at room temperature for 4 h, and the production of pBSTNAV-*serT* plasmid was confirmed by DNA sequencing (Macrogen, Korea), which was used to transform *cmoM*-deficient competent cell (NBRP, Japan) using heat-shock method.

For overexpression of tRNA, *cmoM*-deficient *E. coli* cells were transformed with pBSTNAV-*serT* plasmid and grown in 500 ml LB containing 100 μg/ml ampicillin at 37°C for 24 h. Cells were collected by centrifugation and cellular RNA was extracted by saturated phenol (pH 4.5) (Biosesang, Korea) following the manufacturer's protocol. 3 M sodium acetate (pH 5.3) and isopropanol were added to precipitate RNA, followed by three times of washing with 75% (v/v) cold ethanol. RNA loading dye containing 0.025% (w/v) bromophenol blue and xylene cyanol FF in 95% (v/v) was added to the RNA solution and the mixture was applied to a 7 M urea-PAGE gel, which was run for 14 h at 40 W. The recombinantly overexpressed tRNA^Ser1^ could be resolved from endogenous tRNAs on the gel due to the difference in size. The band containing the desired tRNA was excised by scalpel and tRNA was eluted from the gel strip using Elutrap (Whatman). An extinction coefficient of ϵ_260_ = 852 000 M^−1^ cm^−1^ was used to quantify tRNA^Ser1^. Purified tRNA was denatured at 95°C for 5 min followed by incubation on ice for 20 min before use.

### Crystallization, data collection, and structure determination of CmoM-tRNA^Ser1^(cmo^5^UGA)-sinefungin complex

Crystallization of CmoM-tRNA complex was performed by mixing 1:4 molar ratio of CmoM and *in vivo* transcribed tRNA^Ser1^(cmo^5^UGA) extracted from *cmoM*-deficient cells. Sinefungin (Sigma-Aldrich) was added to the mixture at the final concentration of 3 mM, which was used for co-crystallization of ternary complex of CmoM-tRNA^Ser1^(cmo^5^UGA)-sinefungin by sitting drop vapor diffusion method. An equal volume of the mixture was added to a reservoir solution (0.04 M magnesium acetate tetrahydrate, 0.05 M sodium cacodylate trihydrate (pH 6.0), and 30% (v/v) (+/−)-2-methyl-2,4-pentanediol) at 25°C. Hexagonal prism shaped crystals were formed in 5 days. Crystals were mounted in a nylon loop and flash-cooled in liquid nitrogen. X-ray diffraction data were collected on Dectris Eiger X9M detector at Pohang Accelerator Laboratory (PAL) beamline 5C, using the wavelength *λ* = 0.9795 Å. Structure factors were scaled by XDS (19) and Aimless (20) under space group P6_1_22. To solve the phases of structure factors, molecular replacement was performed with Molrep (21) using the structure of SAM-bound CmoM from *E. coli str. O157:H7* (PDB ID: 4HTF) as a starting model. Extra electron densities corresponding to tRNA were evident after initial refinements with a protein-only model, which guided us to build tRNA component using Modelcraft (22). Subsequent model building and refinement were performed iteratively using Modelcraft (22), Coot (23), PDB REDO (24), REFMAC5 (25), and Phenix Refine (26). Crystallographic statistics are available in Table 1.

**Table 1.** Crystallographic statistics

	CmoM-tRNA^Ser1^(cmo^5^UGA)-sinefungin
**Data collection**	
Wavelength (Å)	0.9795
Space group	*P*6_1_22
Unit cell (*a*, *b*, *c*) (Å) (α, β, γ) (°)	65.614, 65.614, 584.532 90 90 120
Resolution (Å)	40.74 – 2.22 (2.30–2.22)
*R_merge_*	0.2426 (2.553)
*R_meas_*	0.2462 (2.589)
*R_pim_*	0.04123 (0.4229)
Total reflections	1 386 230 (138 756)
Unique reflections	38 928 (3750)
Mean(I)/sd(I)	16.92 (1.52)
Completeness (%)	99.94 (100.00)
Multiplicity	37.2 (37.1)
CC_1/2_	0.928 (0.642)
Wilson *B*-factor (Å^2^)	42.78
**Refinement**	
Resolution range	40.74–2.22
Reflections used in refinement	38920 (3750)
Reflections used for free set	2000 (192)
*R*_work_/*R*_free_	0.1985/0.2252
RMSD bonds (Å)	0.007
RMSD angles (°)	0.96
Ramachandran favored (%)	98.43
Ramachandran allowed (%)	1.57
Ramachandran outliers (%)	0.00
Total number of non-hydrogen atoms	4341
Macromolecules	3956
Ligands	34
Solvent	351
Protein residues	257
Average *B* (Å^2^)	45.50
Macromolecules	39.34
Ligands	33.77
Solvent	44.11

### LC–MS analysis of *in vivo* transcribed tRNA^Ser1^(cmo^5^UGA)

70 μg of tRNA^Ser1^(cmo^5^UGA) was digest into nucleotides by incubating in a solution containing buffer A (50 mM ammonium acetate (pH 6.0), 5 mM ZnCl_2_, and 10 mM MgCl_2_), 1 U of P1 nuclease (Sigma-Aldrich), and 0.1 U of phosphodiesterase I (Sigma-Aldrich) at 37°C for 8 h. For generation of nucleosides, 1 U of FastAP (Thermo Scientific) was added to the above mixture. The nucleotide or nucleoside sample was injected into a Bruker Impact II UPLC-QToF-MS system coupled to a reverse phase HPLC column (Agilent ZORBAX Eclipse Plus C18 Column 95 Å, 3.5 μm, 4.6 mm × 100 mm) for analysis in negative or positive mode, respectively. A gradient step with Solvent A (Water with 0.1% (v/v) formic acid) and Solvent B (Acetonitrile with 0.1% (v/v) formic acid) were applied at a flow rate of 0.4 min/ml as the following; (i) 1–12% of solvent B for 0–17.5 min; (ii) 12–70% of solvent B for 17.5–20 min; (iii) 70% of solvent B for 20–25 min; (iv) 1% of solvent B for 25–55 min. The ESI parameters were set as the following: end plate offset: 500 V, capillary: 4500 V, nebulizer: 4 bar, dry gas: 8 l/min, dry temperature: 200°C. The collision energy was set to 20 eV. MS peaks were extracted from MZmine2 software (27) and plotted using Prism software (version 5.0, GraphPad, USA).

### HPLC based in vitro activity assays of CmoM variants

0.5 μM CmoM was added to the solution containing 25 mM HEPES (pH 7.5), 150 mM NaCl, 50 μM tRNA and 100 μM SAM to initiate enzyme reaction. Reaction mixture was incubated at 37°C for 0–5 min and the reaction was terminated by addition of 0.05% (v/v) formic acid. Sample was injected to a normal phase HPLC column (SeQuent ZIC-HILIC Column, 200 Å, 5 μm, 4.6 × 150 mm) for analysis of SAM and *S*-adenosyl-l-homocysteine (SAH) peaks. A gradient step with Solvent C (5 mM ammonium formate, pH 3.3) and Solvent D (Acetonitrile:50 mM ammonium formate, pH 3.3 = 90:10) were applied at a flow rate of 1 min/ml as the following; (i) 0% of solvent C (100% of solvent D) for 0–8 min; (ii) 0–60% of solvent C for 8–20 min; (iii) 100% of solvent C for 20–25 min; (iv) 0% of solvent C (100% of solvent D) for 25–35 min. The DAD detector was set to 260 nm, 5 Hz.

## RESULTS

### The overall structure of CmoM-tRNA^Ser1^(cmo^5^UGA)-sinefungin complex

**Figure 2.**
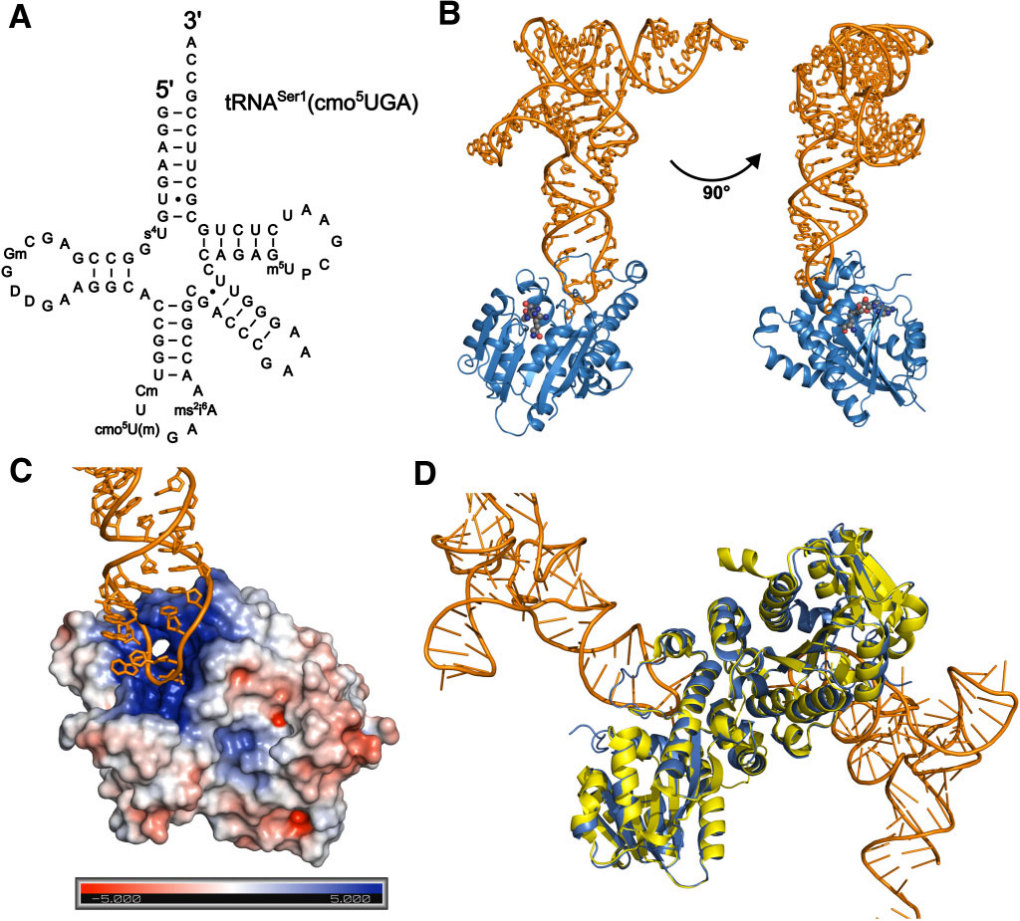
Crystal structure of *E. coli* CmoM-tRNA^Ser1^-sinefungin complex. (**A**) Sequence of cellularly transcribed tRNA^Ser1^(cmo^5^UGA) from Δ*cmoM E. coli* including known modifications, which was used for crystallography and biochemical assays in this study. (**B**) Overall structure of *E. coli* CmoM complexed with tRNA^Ser1^(cmo^5^UGA) and sinefungin. CmoM and tRNA^Ser1^(cmo^5^UGA) are shown in marine and orange ribbons, respectively. Sinefungin is represented in sphere, where carbon is shown in grey, oxygen in red, and nitrogen in blue. (**C**) Docking site of tRNA (orange) on CmoM is shown, where electrostatic potential is mapped on the surface of the enzyme (positive in blue, negative in red, and neutral in white). (**D**) Superposition of reconstituted dimer of CmoM-tRNA complex (marine and orange for CmoM and tRNA, respectively) and tRNA-free CmoM (PDB ID: 4htf, yellow) structures.

To investigate the molecular interaction between the enzyme and the hypomodified base, cmo^5^U34, we employed recombinantly expressed and purified tRNA^Ser1^ from c*moM*-deficient *E. coli* mutant for structural study (Figure 2A). In addition, an analog of SAM, sinefungin, was used for co-crystallization of the ternary complex to mimic a substrate-bound state of CmoM. The X-ray co-crystal structure of CmoM-tRNA^Ser1^(cmo^5^UGA)-sinefungin was determined to 2.22 Å resolution (Figure 2B). The asymmetric unit of the X-ray crystal structure contains a tRNA molecule bound to a protomer of CmoM, mainly through the anticodon loop on the protein surface enriched with positive charges (Figure 2C). The previously reported SAM-bound structure (PDB ID: 4HTF) displays a dimeric form of the enzyme and an essentially identical dimer can be generated with a symmetry-related protomer from our structure (Figure 2D). We examined the oligomeric state of CmoM via size-exclusion chromatography, where the results were consistent with the dimeric form (Supplementary Figure S1). Analysis of the dimeric interface of the protein reveals that the dimerization is mainly driven by polar interactions involving a total of eight residues from each protomer (Supplementary Figure S2) (28). Overall, the residue interactions at the dimer interface in our complex structure are essentially identical to those in the SAM-bound structure. In detail, the sidechain of R234 interacts with the carbonyl oxygen of Q169, while the imidazole ring of H153 interacts with the hydroxyl group of Y241. The remaining eight residues form a complex hydrogen bond network at the core of the interface. The sidechain of R216 interacts with the backbone carbonyl oxygens from both D213 and L215, whereas the backbone amide group of R216 interacts with the carbonyl oxygen of V161.

CmoM is a member of Class I SAM-dependent methyltransferases (MTases), which adopts a Rossmann-fold with a β-sheet architecture of 3–2–1–4–5–7–6, where the 7th β-strand lies antiparallel to other β-strands (Supplementary Figure S3) (29). Ten α-helices flanking the β-sheet form a sandwich-like αβα-fold as observed in numerous Class I MTases (30). Other characteristic features of this family include the presence of conserved elements involved in SAM binding, such as the glycine-rich motif at the β1 (G52–G53–G54) and the amino acid residues D73 at the end of β2 and R26 at the first one-third position of α2, which interact with the ribose hydroxyl groups and the methionine carboxylate, respectively. Other residues such as D3, A101, and Q102 recognizes SAM’s adenine whereas Y18 interacts with the methionine carboxylate (Supplementary Figure S4). The search for structural homologs with the DALI server (31) yielded an ubiquinone/menaquinone biosynthesis methyltransferase-related protein from *Thermotoga maritima* (PDB ID: 2AVN) as a top hit, with an z-score of 25.9 and a root-mean-square deviation (r.m.s.d.) of 2.7 Å, which shares a sequence identity of 21% with CmoM over 248 residues (Supplementary Figure S5).

The overall conformation of tRNA-complexed CmoM observed in our model is not significantly different from that of the tRNA-free state, where the superposition of both structures results in an r.m.s.d. of 0.450 Å. Moreover, the microenvironment around sinefungin is highly homologous to that of the SAM binding site in the tRNA-free structure, where sinefungin fits identically as the conformation of SAM. Notably, a total of seven Mg^2+^ sites were identified around tRNA in our model. Only two of those are directly coordinated to the phosphate oxygen of C48 and A59, whereas the others are in contact with tRNA molecule indirectly through water shell (Supplementary Figure S6).

### tRNA recognition by CmoM

**Figure 3.**
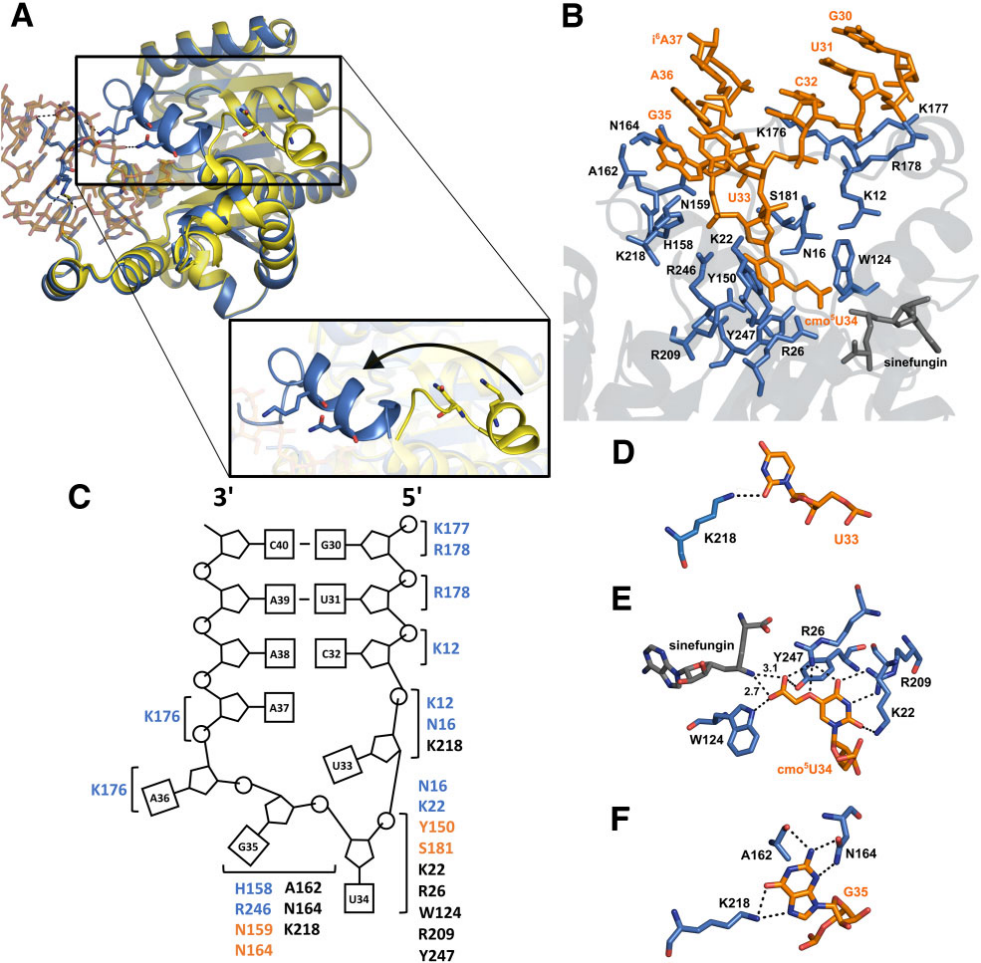
Intermolecular interactions between CmoM and tRNA. (**A**) Superposed structures of tRNA-complexed (marine) and tRNA-free (PDB ID: 4htf, yellow) CmoM, where the inset highlights a large shift of α1 helix upon binding of tRNA. (**B**) Active site residues of CmoM (blue) that interact with tRNA anticodon loop region (orange). Sinefungin is shown in grey sticks. (**C**) A schematic diagram tRNA showing polar interactions with the enzyme at each residue, where the phosphate is represented by circle, ribose by pentagon, and base by square. Amino acids associated with the phosphate backbone (blue), sugar (orange), and base (black) are grouped in brackets for individual nucleotides. Amino acid residues interacting with nucleobase of (**D**) U33, (**E**) cmo^5^U34 and (**F**) G35 are shown, where dashed lines represent hydrogen bonding interaction. Distance between the amine of sinefungin and the carboxyl group of cmo^5^U34 is labeled in Å.

There are a couple of substantial changes in local conformation induced by the tRNA-binding (Figure 3A). The N-terminal helix (α1) swings towards the bound tRNA by approximately 30 Å to establish contact with phosphate groups of C32 and U33. Another notable change is observed in the organization of the loop encompassing residues 170–188, which is mostly disordered in the tRNA-free structure. In the tRNA-bound state, this loop mainly interacts with the phosphates of G30, U31, A36, and i^6^A37 through the formation of salt bridges. All in all, eight nucleotides of tRNA (G30 through i^6^A37) are in direct contact with CmoM, six of which are located on the anticodon loop (Figure 3B,C). A total of 18 amino acid residues are found at the interface with tRNA, where eight of those sense the shape of the phosphate backbone on the anticodon loop through making salt-bridges or hydrogen bonds; i.e. K177 with G30, R178 with G30 and U31, K12 with C32 and U33, N16 with U33 and cmo^5^U34, K22 with cmo^5^U34, H158 and R246 with G35, and K176 with A36 and i^6^A37. Four amino acid residues make contact with the ribose ring of cmo^5^U34 and G35 by forming hydrogen bonds; i.e. the sidechains of Y150 and S181 with the 2′-hydroxyl group of cmo^5^U34, whereas those of N159 and N164 with the O4’ of G35.

Only three nucleobases of the entire tRNA molecule, U33, cmo^5^U34, and G35, participate in ionic or polar interactions with the protein. U33 appears to interact solely with K218 by forming a hydrogen bond via the O2 of uracil (Figure 3D). Meanwhile, the hypomodified base of cmo^5^U34, which has flipped out from the canonical stacking position, exhibits the most extensive interactions with the enzyme (Figure 3E). A total of five residues are involved in multiple ionic/polar interactions with the modified uracil base, where the 5-carboxymethoxy group is thoroughly examined by R26, W124 and Y247. Notably, R26 of CmoM forms a salt bridge and hydrogen bond with the carboxyl and ether oxygen, respectively. Furthermore, the carboxyl group on the modified uracil base engages in a hydrogen bonding network with the side chains of W124 and Y247. Therefore, these three residues appear to be primarily responsible for the proper positioning of the nucleophilic carboxyl group on cmo^5^U34, which is approximately 3 Å away from the Nϵ of sinefungin. Lastly, K22 interacts with O2, and R209 interacts with N3 and O4 of cmo^5^U34. Meanwhile, the guanine base of G35 interacts with three amino acid residues (Figure 3F). The backbone carbonyl oxygen of A162 is within hydrogen bonding distance from N1 and N2. N164 forms hydrogen bonds with N2 and N3 via its sidechain, while K218 interacts with O6 and N7.

### Biochemical analyses of key amino acid residues in tRNA binding and catalysis

**Figure 4.**
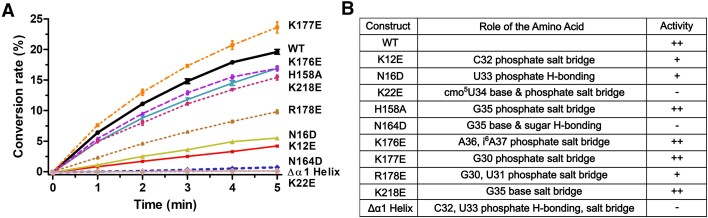
Mutagenesis studies of amino acid residues participating in ionic/polar interactions with tRNA. (**A**) The conversion rates of cmo^5^U to mcmo^5^U by the wild-type and mutant CmoM are plotted, which have been determined through *in vitro* assays using SAM and tRNA^Ser1^ extracted from *E. coli ΔcmoM* cells. Error bars represent the standard deviation from three independent measurements at each time point. (**B**) Roles of amino acid residues selected for mutagenesis and relative activities of mutants are summarized, where ++ stands for the wild-type comparable, + for moderate and – for minimal activity.

To verify the functional role of the amino acid residues that were identified to interact with tRNA in our structure, site-directed mutagenesis was performed to introduce a single mutation and the biochemical activity of the mutant proteins was examined (Figure 4). Previously, Suzuki's group tested the biosynthesis of mcmo^5^U in tRNA^Pro3^ from *cmoM-*deficient strain complemented with plasmid-encoded *cmoM*-variants harboring an alanine mutation at R26, D73, W124, Y150, R209, D213, R246 and Y247, which were identified around the SAM-binding site in the tRNA-free structure (17). Therefore, these residues were excluded from our investigation. Moreover, we followed the time-course of the accumulation of SAH, a coproduct of the methyltransfer reaction, to quantitatively measure the *in vitro* activity of the enzyme using recombinantly purified CmoM and tRNA^Ser1^ containing cmo^5^U at the wobble position.

The assay results highlight the importance of N164, which, along with A162 and K218, is one of the three residues recognizing G35 base. When mutated to aspartate, the mutant enzyme showed a sever diminishment in methyl transfer activity. Additionally, K22 also appears to be critical in our assay, which forms a hydrogen bond with the base and salt bridge with the phosphate of cmo^5^U34. Mutation of this residue to glutamate resulted in total inactivation, suggesting its importance in identifying the flipped-out base of cmo^5^U34. Other notable residues include K12 and N16, as the mutation of each led to a substantial decrease in activity, nearly 80% lower than that of the wild-type enzyme. Interestingly, both of these residues are located on the N-terminal helix, α1, which was observed to undergo a movement towards tRNA upon binding as shown in the structure. When we tested a mutant CmoM lacking α1, the truncated enzyme was completely inactive. This suggests that the binding of tRNA had been seriously compromised, underscoring the essential roles of the N-terminal helix. The other secondary structure that displays substantial conformational rearrangement induced by tRNA docking is the loop composed of amino acid residues from 170 to 188. Within this loop, K176, K177 and R178 are identified as interacting with the phosphate backbone of the tRNA substrate through the formation of salt bridges. While K176E and K177E did not substantially affect the enzyme activity, R178E resulted in an approximate 50% decreased activity compared to the wild-type. Meanwhile, the alanine mutant of H158, which interacts with the phosphate of G35, displays a negligible effect on methylation activity. Similarly, when K218, which interacts with both U33 and G35 bases, was altered to glutamate, the activity of the mutant was comparable to that of the wild-type. Interestingly, K218 is the only residue in the structure that contacts the U33 base, suggesting that U33 is not a crucial determinant for CmoM.

### Conformation of full-length tRNA^Ser1^

**Figure 5.**
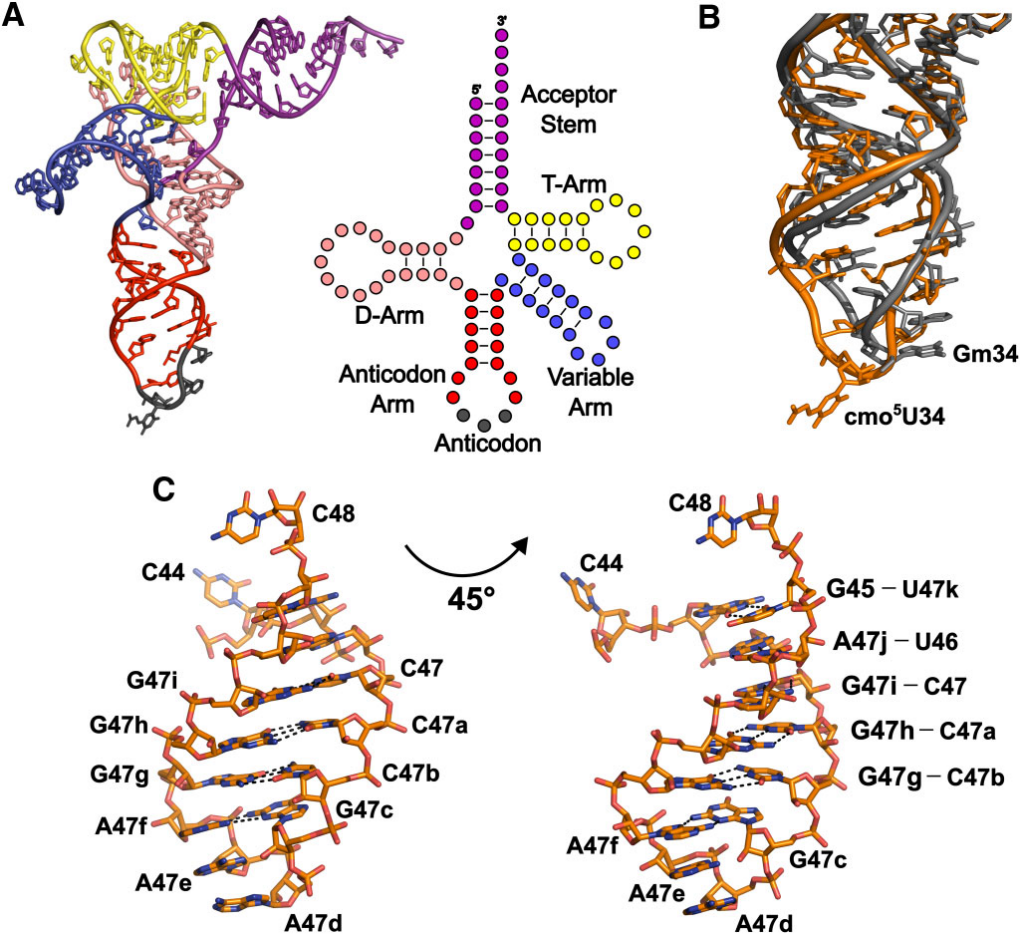
Features of cellularly transcribed tRNA^Ser1^. (**A**) Structure of the CmoM-bound tRNA with individual regions shown in different colors; acceptor stem in purple, D-arm in salmon, anticodon arm in red (anticodon in dark grey), variable arm in blue, and T-arm in yellow. (**B**) Superposition of anticodon stem loops of tRNA^Ser1^ (orange) and *S. cerevisiae* tRNA^Phe^ (PDB ID: 1EHZ, grey), which highlights the conformational change in tRNA^Ser1^, especially the flipping-out cmo^5^U34. (**C**) A close-up view of the long variable arm of tRNA^Ser1^. Base pairing interactions are represented in black dashed lines.

All 88 nucleotides in tRNA^Ser1^ could be clearly modeled in the present structure (Figure 5A and Supplementary Figure S7). To our knowledge, this represents the first 3-D structure of a cellularly transcribed, full-length tRNA^Ser1^. The most notable feature observed in the CmoM-bound tRNA conformation is the rearrangement of the anticodon loop region, particularly the flipping out of cmo^5^U34, whereas anticodon bases are in general stacked on one another as observed in other free- or ribosome-bound tRNA structures (32–34) (Figure 5B). Furthermore, our tRNA structure exhibits an atypical U-turn motif within the anticodon loop, where two characteristic hydrogen bonds required for the formation of the anticodon U-turn (35) are not observed; *i.e*. the interaction between N3 of U33 and the phosphate of N36, as well as that between the 2′-OH of U33 and the nucleobase of N35. This deformation of the anticodon loop is likely a result of the accompanying flip-out of cmo^5^U34. Another interesting structural feature of the CmoM-bound tRNA^Ser1^ is the presence of a long variable arm composed of 16 nucleotide residues; C44, G45, U46, C47, C47a, C47b, G47c, A47d, A47e, A47f, G47g, G47h, G47i, A47j, U47k, and C48 (Figure 5C). This extended variable arm is significantly longer than those found in other tRNA species, which are typically composed of 4–5 nucleotides (36). The variable arm is well-defined in our structure mainly due to the lattice contacts provided by symmetry-related molecules at the unit cell interface. The stem region of the variable arm is composed of four Watson-Crick (U46-A47j, C47-G47i, C47a-G47h and C47b-G47g) and two non-canonical (G45-U47k and G47c-A47f) base pairs, which is capped with a short turn composed of four nucleotides (G47c to A47f).

### Native tRNA modifications

Because we used cellularly derived tRNA for crystallization, we attempted to model all known post-transcriptional modifications in the structure. There are seven different types of modified nucleotides in *E. coli* tRNA^Ser1^ in addition to the hypermodified wobble uridine (37); e.g. 4-thiouridine (s^4^U8), 2′-*O*-methylguanosine (Gm18), dihydrouridine (D20 and D20a), 2′-*O*-methylcytidine (Cm32), 2-methylthio-N^6^-isopentenyladenosine (ms^2^i^6^A37), 5-methyluridine (m^5^U54), and pseudouridine (P55). The presence of certain modifications was evident in the *F*_o_ – *F*_c_ differential Fourier density map, especially bulky ones such as 5-caroxymethoxy moiety on U34 and *N*^6^-isopentenyl group on A37 (Supplementary Figure S8). Interestingly, we could not model 2-methylthio group on A37 because of the complete lack of electron density, even though this residue is known to contain both N^6^-isopentenyl and 2-methylthio moieties (ms^2^i^6^A). Meanwhile, it was generally ambiguous to model relatively small-sized modifications based on the electron density with the given resolution, for example, D, Cm, Gm and P.

To examine whether the tRNA sample contains all the expected modifications, we analyzed the content of the recombinant tRNA^Ser1^ using mass spectrometry. The tRNA sample was digested to nucleosides or nucleotides and then subjected to ultra performance liquid chromatography (UPLC) coupled with electrospray ionization mass spectrometry. To obtain a comprehensive profile of posttranscriptional modifications present in the sample, the nucleosides were analyzed in positive mode, while the nucleotides containing a 5′-phosphate were analyzed in negative mode. Pseudouridine was excluded from our analyses as it cannot be distinguished from uridine through simple mass spectrometry. We confirmed the presence of the 5′-monophosphates of s^4^U, D, cmo^5^U and m^5^U in negative mode, while Gm, Cm and ms^2^i^6^A were observed in positive mode (Supplementary Figure S9). Surprisingly, we also detected N^6^-isopentenyladenosine (i^6^A) in both positive mode, likely as a precursor of ms^2^i^6^A at position 37, and negative mode as its 5′-monophosphate form. To determine the relative abundance of tRNA species containing ms^2^i^6^A or i^6^A, the intensities of the characteristic fragments, N^6^-isopentenyladenine and 2-methylthio-*N*^6^-isopentenyladenine, were measured using multiple reaction monitoring (MRM) mode analysis. Based on the comparison of peak areas for each modified base, it is estimated that i^6^A-containing species are approximately 5.3-times more abundant than ms^2^i^6^A-containing one (Supplementary Figure S10).

## DISCUSSION

**Figure 6.**
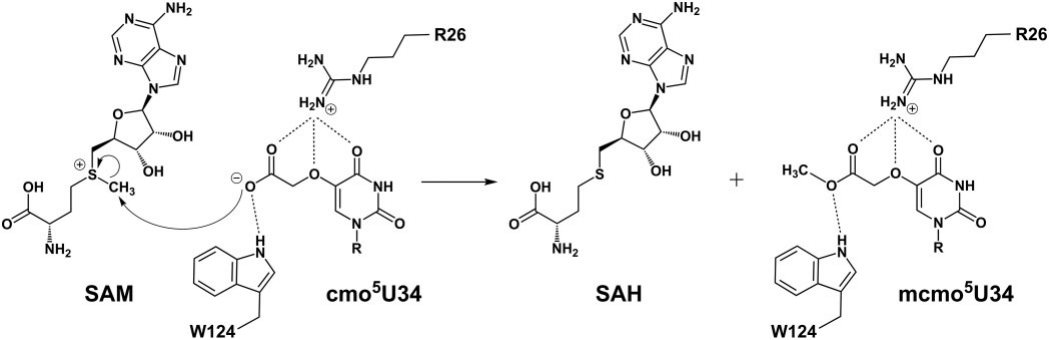
A proposed reaction mechanism for the SAM-dependent cmo^5^U methylation by CmoM. Kinetically essential residues such as R26 and W124 are positioned to fine-tune the orientation of the nucleophile and prevent the binding of the protonated form to promote the nucleophilicity. Subsequently, the carboxylate of cmo^5^U34 attacks the *S*-methyl group of SAM to form mcmo^5^U34 and SAH.

The most critical determinant on tRNA for recognition by CmoM is cmo^5^U34. In the structure, the calculated buried surface area of this nucleotide alone accounts for approximately 30% of the total interface between the protein and tRNA (317.5 out of 1072.6 Å^2^). It is plausible that the flipped-out hypomodified base from the canonical stacked conformation is stabilized by the extensive intermolecular interactions with eight amino acid residues from the enzyme. Notably, R26, W124 and Y247 appear to fine-tune the orientation of the nucleophilic carboxymethoxy group on the wobble uridine to facilitate the efficient methyl transfer step. Indeed, the previous mutagenesis study showed that the enzyme activity is abolished with R26A and W124A, or partially decreased with Y247A (17). Due to its low pKa value, it is likely that the carboxymethoxy group does not require a general base to become an efficient nucleophile during methyltransfer reaction, which is further ensured by the ionic/polar interactions with those residues (Figure 6). In *E. coli*, it has been demonstrated that tRNA^Ala1^, tRNA^Ser1^, tRNA^Pro3^ and tRNA^Thr4^, which contain mcmo^5^U34, have guanosine at position 35, while tRNA^Leu3^ and tRNA^Val1^, which contains cmo^5^U34, have adenosine at the corresponding position. Therefore, G35 is likely an important determinant for CmoM in distinguishing its substrates, although a low-level mcmo^5^U formation in tRNA^Leu3^ and tRNA^Val1^ has been reported at a frequency below 10% (17). Our structure of CmoM-tRNA^Ser1^(cmo^5^UGA)-sinefungin complex provides supporting evidence by unveiling detailed information on how the enzyme identifies the correct base at this position. The exocyclic amine group at C2 of guanine is one of the key structural features compared to adenine, which is specifically recognized by the amido sidechain of N164 and the backbone carbonyl group of A162. Our mutagenesis experiments further emphasize the importance of G35 recognition by N164, as the activity of N164E was almost completely abolished.

The extended variable arm is a characteristic feature of class II tRNAs, including tRNA^Ser^, tRNA^Leu^, tRNA^Sec^ and tRNA^Tyr^ (38). However, structural information on these tRNAs with a long variable arm is quite limited. Our co-crystal structure features the full-length tRNA^Ser1^ containing a 16 nt-long variable arm, although it is irrelevant to the function of CmoM. Meanwhile, it was shown that a certain stem length of the variable arm is required for recognition by seryl-tRNA synthetase (SerRS) (39). Likewise, the long variable arm of tRNA^Sec^ is crucial for the serine ligation activity of SerRS (40). Structural comparison of these tRNAs reveals an overall similarity, while the variable arm of *E. coli* tRNA^Ser1^ resembles that of *Aquifex aeolicus* tRNA^Sec^ (PDB ID: 3W3S) more than that of *Thermos thermophilus* tRNA^Ser^ (PDB ID: 1SER), both of which are complexed with SerRS (Supplementary Figure S11). Structural and functional characterization of more diverse class II tRNAs will be valuable in understanding the conserved roles of long variable arms, which confer a conformational diversity to the canonical L-shaped scaffold.

Our co-crystal structure shows both expected and unexpected posttranscriptional modifications present in tRNA. Surprisingly, the structural data was consistent with i^6^A at position 37 instead of the expected ms^2^i^6^A. Analysis of hydrolyzed tRNA sample used for structure determination indicates that it is a mixture of both with a relative abundance of 5.3:1. In the biosynthesis of ms^2^i^6^A, MiaA and MiaB are responsible for attaching isopentenyl moiety at N6 and methylthio group at C2 of adenine ring, respectively. The cause of incomplete ms^2^i^6^A formation in our sample is unknown. One possibility is that it resulted from an artifact of using a robust overexpression system, where massive production of recombinant tRNA occurs under the strong promotor of the expression plasmid, while the rate of MiaB-dependent methylthiolation does not increase comparably. An alternative scenario is the potential non-constitutive nature of methylthiolation at A37, although it has not been reported whether MiaB-dependent modification is inducible or not. Further investigation into the cellular and biochemical regulation of MiaB will be required to address the validity of this hypothesis.

## Supplementary Material

gkad668_Supplemental_File

## Data Availability

The atomic coordinates and structure factors for *E. coli* CmoM-tRNA^Ser1^(cmo^5^UGA)-sinefungin are deposited to Protein Data Bank (PDB) under accession code 8JOZ.
