# Supplementary material for: Structural basis for the selective methylation of 5-carboxymethoxyuridine in tRNA modification
Source: Nucleic Acids Res. 2023 Aug 17;51(17):9432–41. doi: 10.1093/nar/gkad668 (PMC10516636; doi:10.1093/nar/gkad668)
Supplement: gkad668_Supplemental_File [file gkad668_supplemental_file.docx]

**Supplementary figures and tables**

**Structural basis for the selective methylation of 5-carboxymethoxyuridine in tRNA modification**

Jaehun Yoo^1^, Jangmin Lee^1^, and Jungwook Kim^1*^

^1^ Department of Chemistry, Gwangju Institute of Science and Technology, Gwangju, 61005, Korea.

* To whom correspondence should be addressed. Tel: +82-62-715-4622; Fax: +82-62-715-2866; e-mail: jwkim@gist.ac.kr

**Primers Used for CmoM *in vivo* Mutagenesis**

| Construct | Forward Primer (5’-3’) | Reverse Primer (5’-3’) |
| --- | --- | --- |
| K12E | CAGGATCGCAATTTTGATGATATTGCGGAAGAGTTTTCCCGTAACATTTACGGC | TTCCGCAATATCATCAAAATTGCGATCCTG |
| N16D | TTTGATGATATTGCGGAAAAGTTTTCCCGTGACATTTACGGCACCACCAAA | ACGGGAAAACTTTTCCGCAATATCATCAAA |
| K22E | TCCCGTAACATTTACGGCACCACCGAAGGGCAGCTTCGACAGGCT | GGTGGTGCCGTAAATGTTACGGGA |
| H158A | TTAATGTTCTACAATGCGCATGGTTTGTTGATGGCTAACATGGTCGCCGGGAATTTT | CATCAACAAACCATGCGCATTGTAGAACATTAA |
| N164D | TTGTTGATGCATAACATGGTCGCCGGGGATTTTGATTACGTGCAGGCG | CCCGGCGACCATGTTATGCATCAACAA |
| K176E | AATTTTGATTACGTGCAGGCGGGAATGCCGAAAAAGGAAAAACGGACGCTTTCGCCA | CTTTTTCGGCATTCCCGCCTGCACGTAATCAAAATT |
| K177E | TACGTGCAGGCGGGAATGCCGAAAAAGAAAGAACGGACGCTTTCGCCA | TTTCTTTTTCGGCATTCCCGCCTGCACGTA |
| R178E | CAGGCGGGAATGCCGAAAAAGAAAAAAGAAACGCTTTCGCCAGATTATCCACGC | TTTTTTCTTTTTCGGCATTCCCGCCTG |
| K218E | CGCGTGTTTCATGATTATCTGCGCGAGGAACACCAGCAGCGC | CTCGCGCAGATAATCATGAAACACGCG |
| Δα1 Helix | ACCATACGGATCCGGCCCTGAGGGCCACCACCAAAGGGCAGCTTCGA | GGCCCTCAGGGCCGGATCCGTATGGTGATG |

**Primers Used for tRNA *in vivo* Transcription Plasmid Preparation**

| tRNA | Forward Primer (5’-3’) | Reverse Primer (5’-3’) |
| --- | --- | --- |
| tRNA^Ser1^(TGA) | GGAAACAGCTATGACCATGATTACGCC | CCATTCAGGCTGCGCAACTGTTGG |

**Supplementary Table 1.** Used primers for PCR.


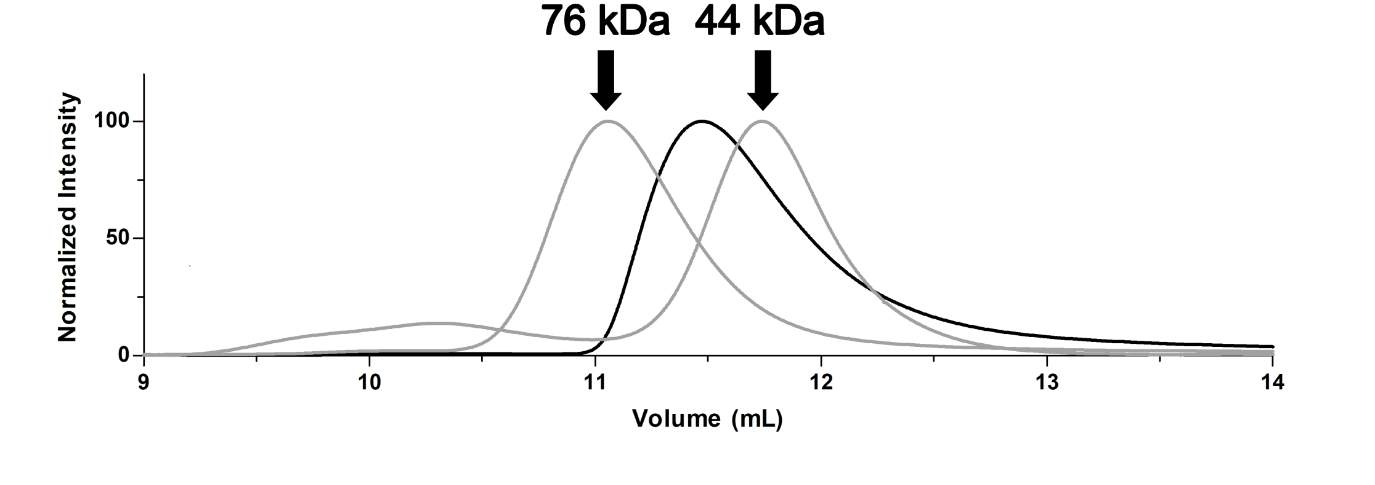


**Supplementary Figure 1. A representative result from size exclusion chromatography of CmoM.** CmoM (black) elutes between MnmC (76 kDa) and ovalbumin (44 kDa) (grey) indicating CmoM forms dimer in solution (Theoretical molecular weight of dimeric CmoM = 63.4 kDa).


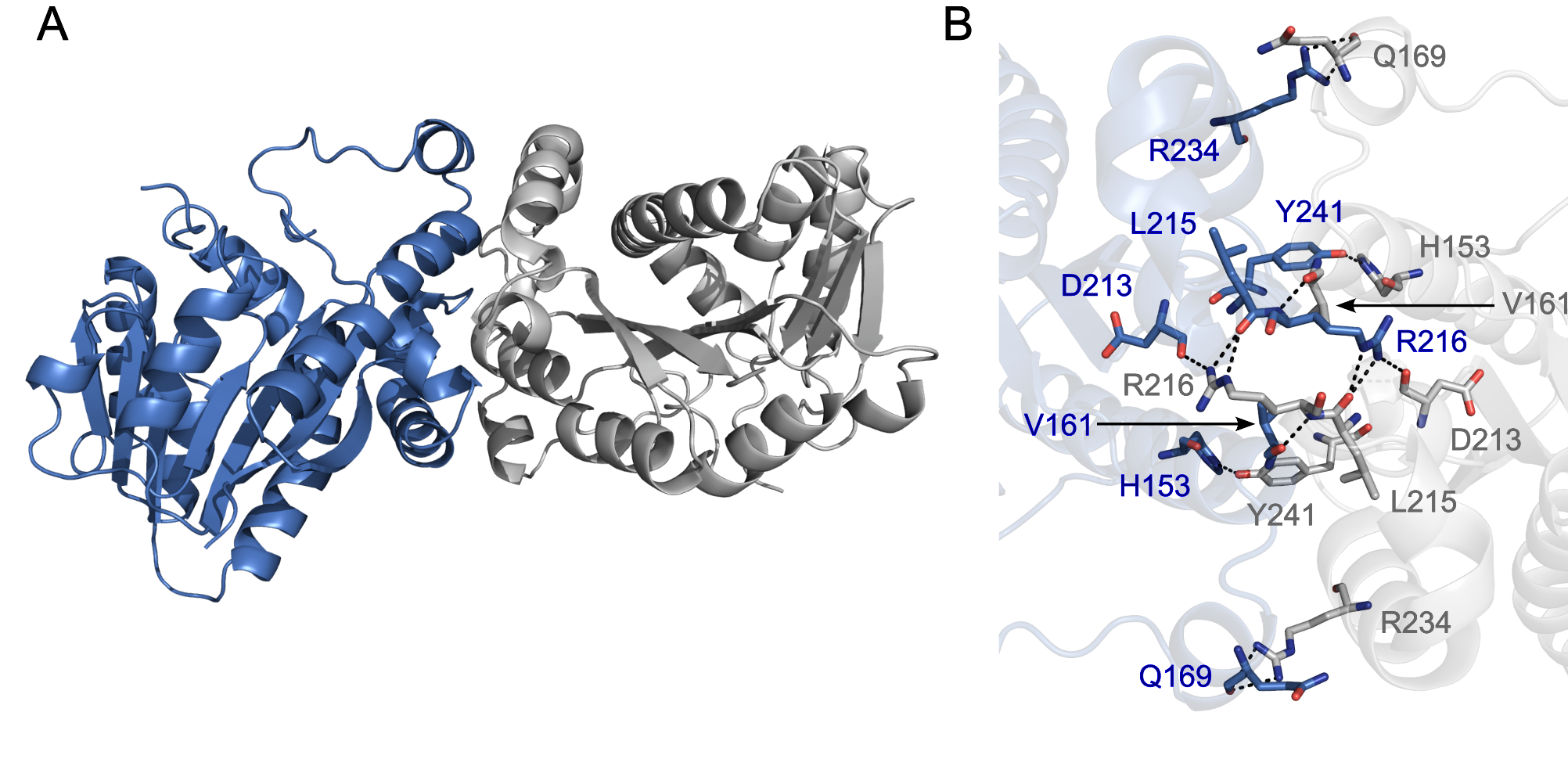


**Supplementary Figure 2.** **Dimeric interface of CmoM.** Each protomer is depicted in marine and grey, respectively. The dimeric interface of CmoM is centered in **A**), where the molecular interactions between amino acid residues are highlighted in a close-up view in **B).** Hydrogen bonds are depicted in black dotted lines.


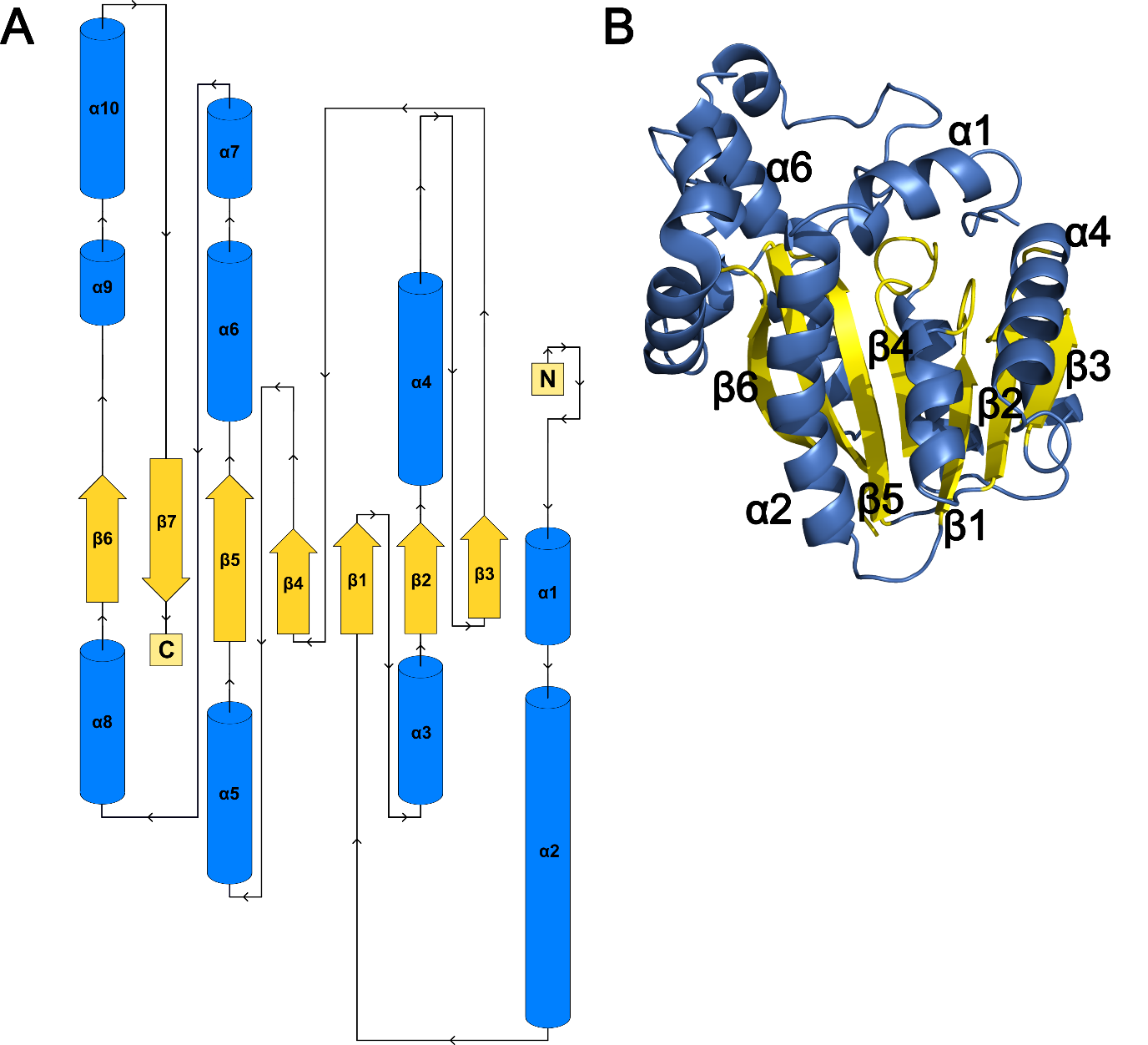


**Supplementary Figure 3.** **Analysis of CmoM topology.** The β-strands and α-helices composing the Rossmann-fold motif are shown in yellow and blue, respectively. **A**) A topology diagram of CmoM. **B**) Ribbon representation of CmoM with labelled secondary structural elements.


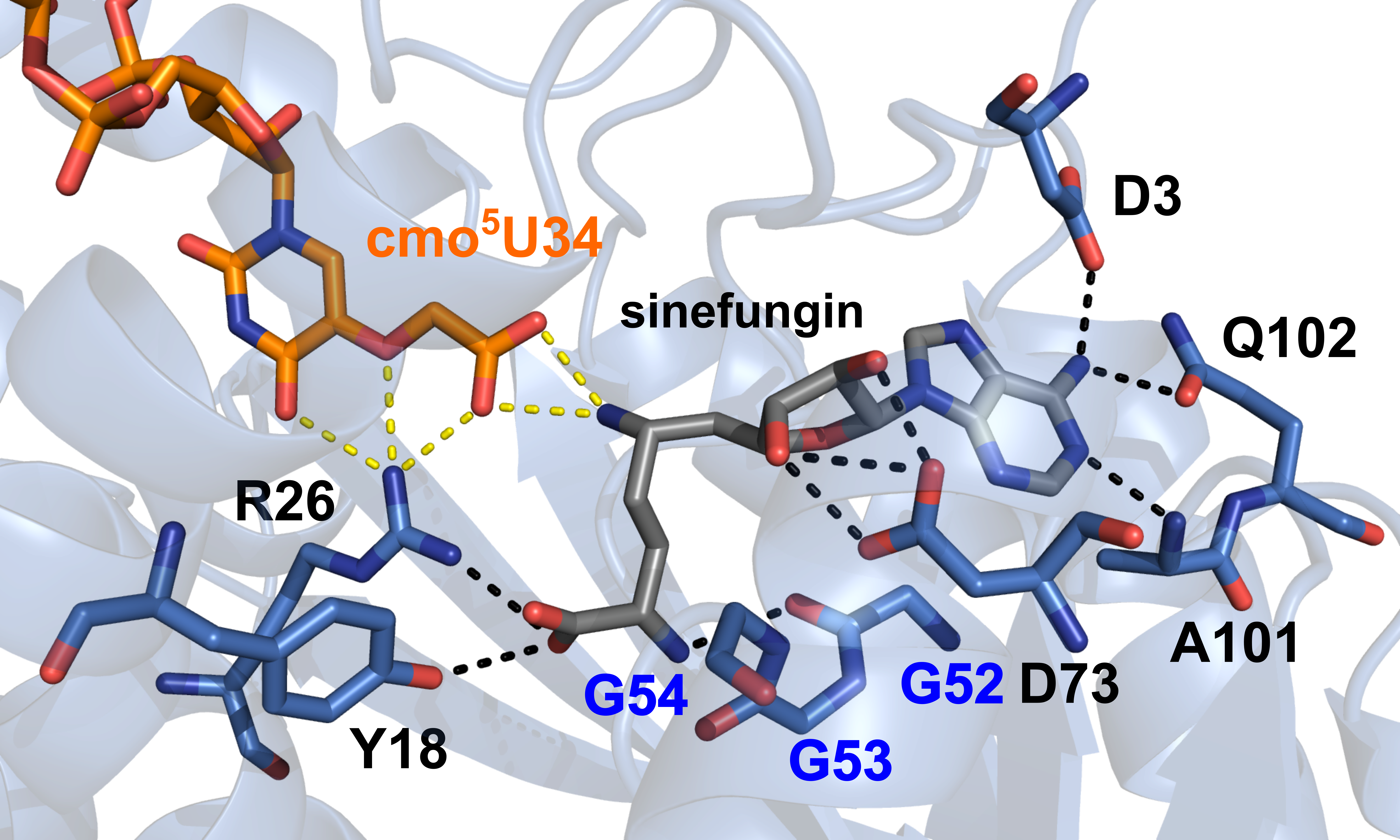


**Supplementary Figure 4.** **Conserved cofactor-binding site.** The residues that are responsible for recognizing sinefungin, a SAM analog, are displayed in sticks. Interactions between amino acids and sinefungin are shown in black dotted lines, while those with cmo^5^U34 are shown in yellow ones. The glycine-rich motif (G52-G53-G54) is highlighted in blue.


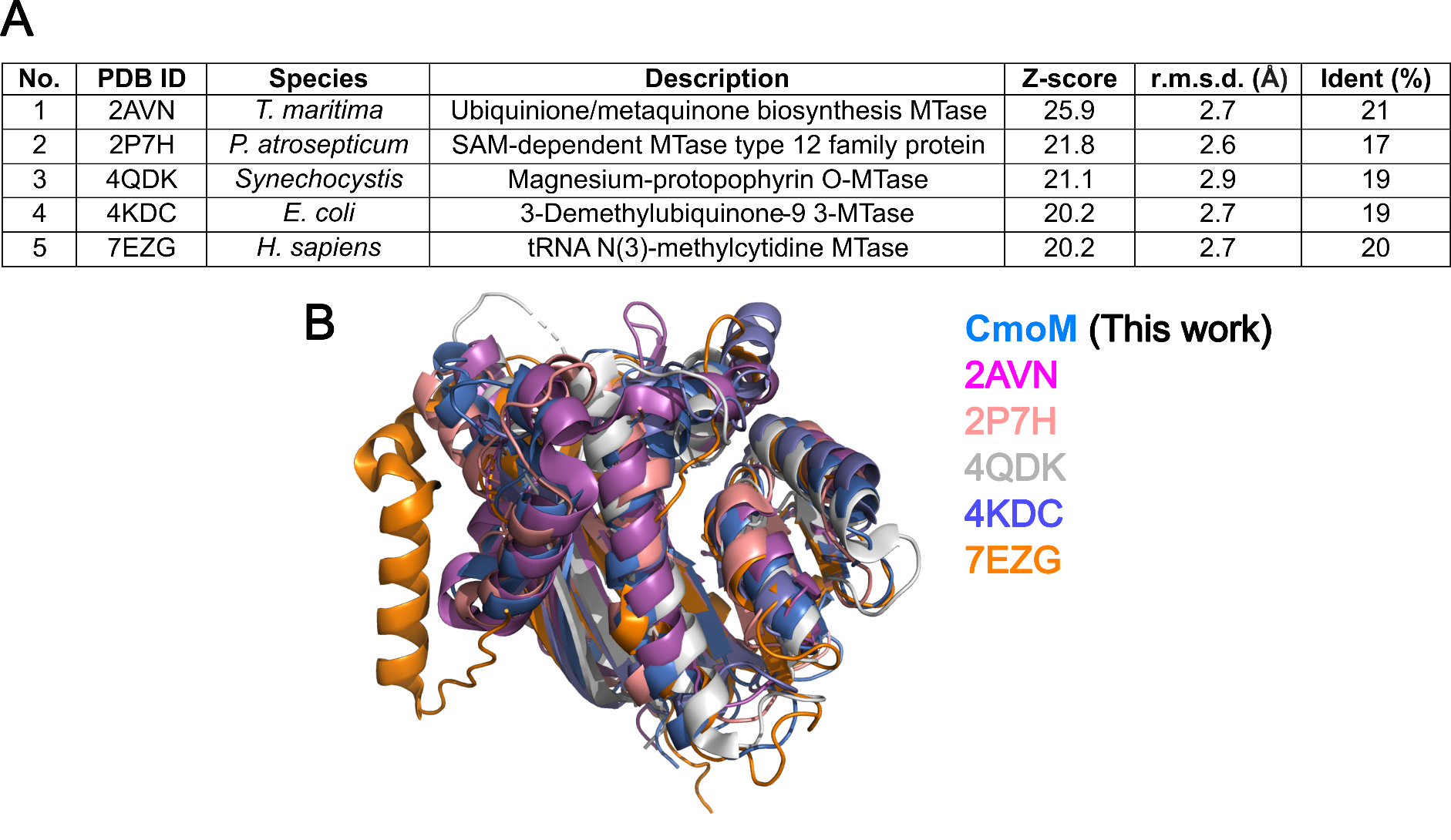


**Supplementary Figure 5.** **Homology search for CmoM.** **A**) The top 5 hits from the homology search of CmoM by DALI server. **B**) Structures of top 5 homologs are overlapped with that of CmoM.

**
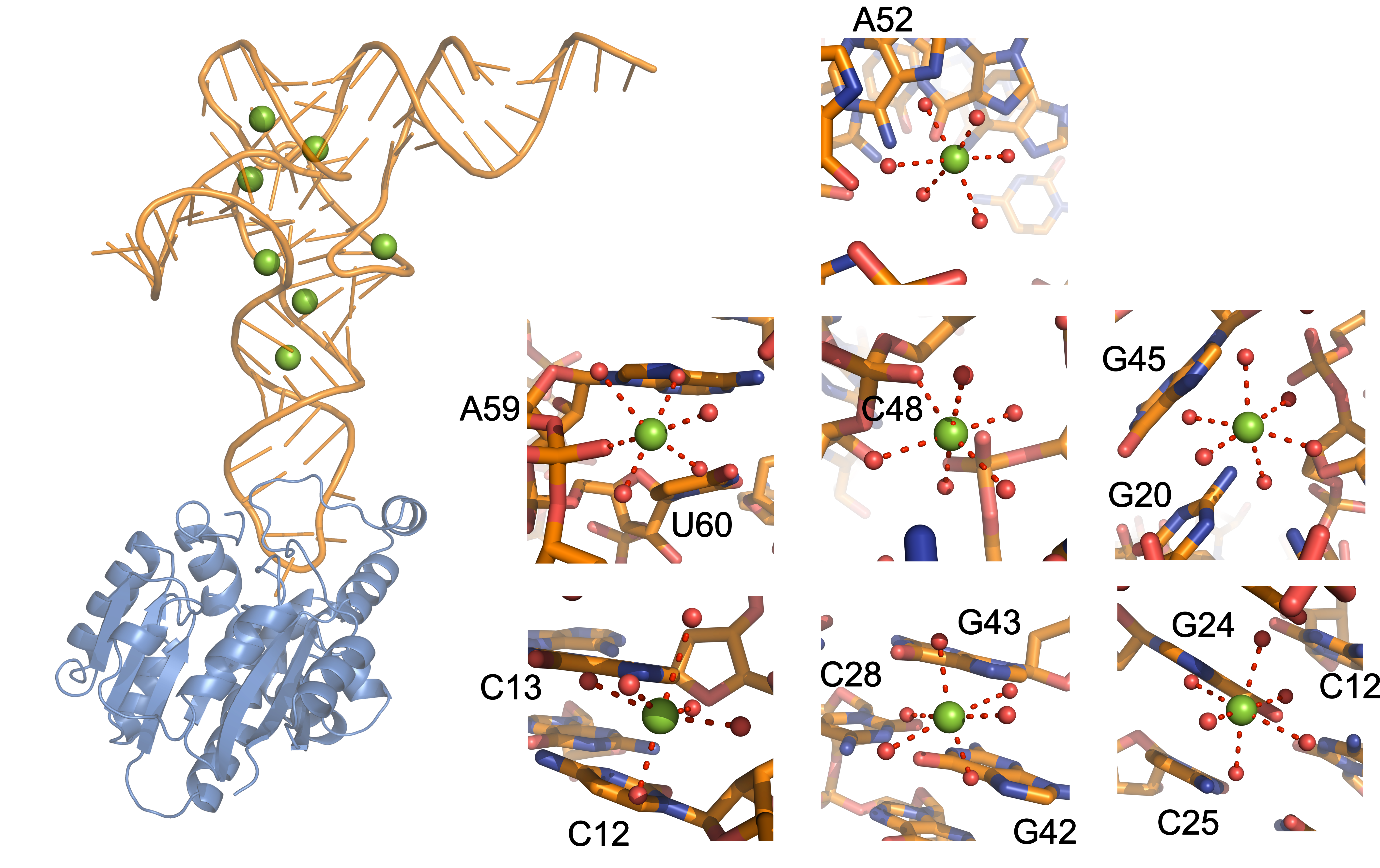
**

**Supplementary Figure 6. Magnesium binding sites**. A total of seven magnesium binding sites were assigned based on the bond lengths and the octahedral geometry of coordination sphere. Magnesium (green) and water (red) are depicted in spheres.


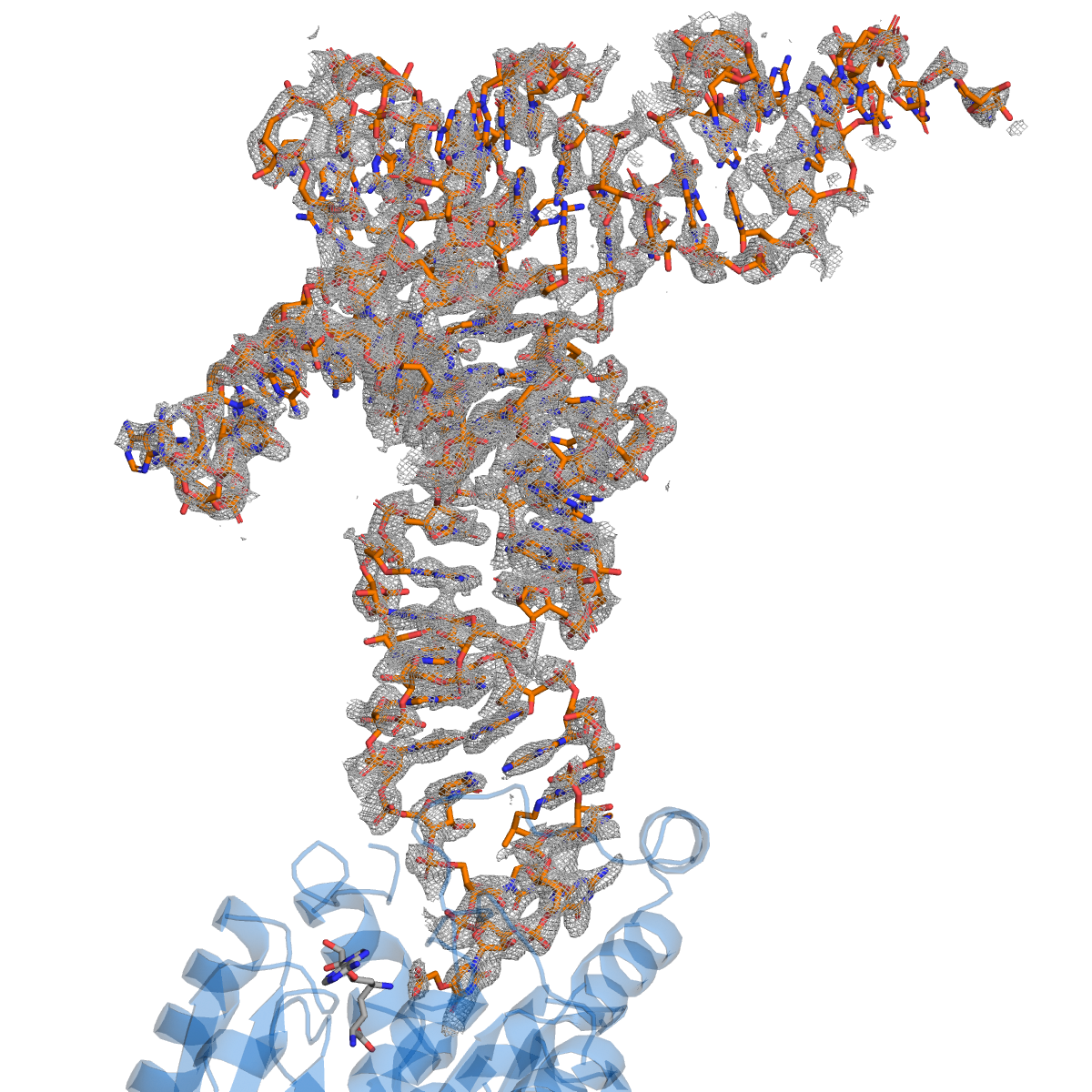


**Supplementary Figure 7.** **Omit electron density map of tRNA^Ser1^.** Fourier difference map (*Fo-Fc*) contoured at 2σ is displayed in grey mesh calculated without tRNA in the model during refinement. CmoM and sinefungin are displayed as transparent marine ribbon and sticks, respectively.

**
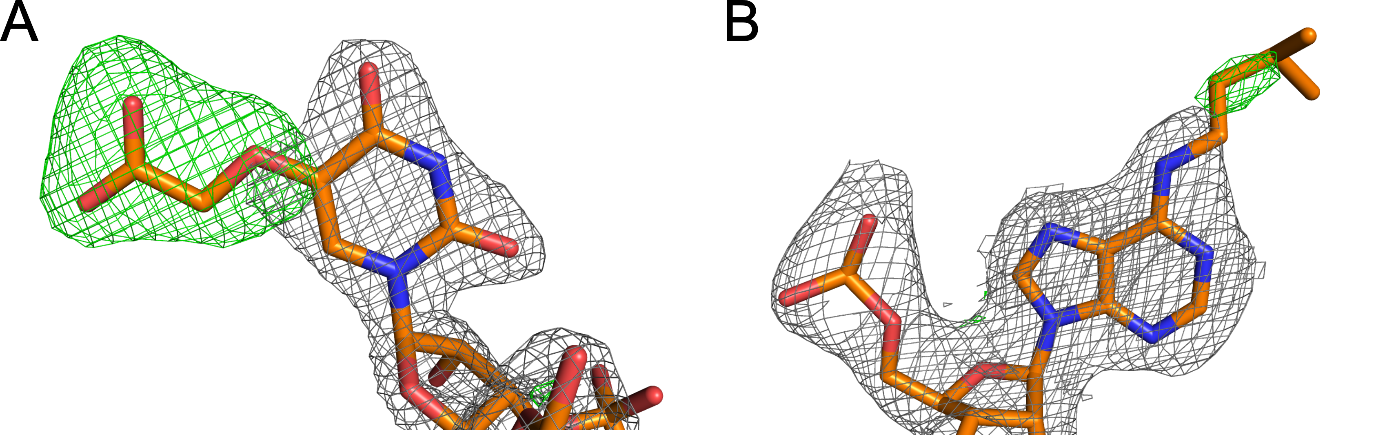
**

**Supplementary Figure 8.** **Omit maps of modified residues A) cmo^5^U34 and B) i^6^A37**. Fourier difference maps of *2Fo-Fc* are displayed in grey mesh (contoured at 2σ) and those of *Fo-Fc* in green mesh (contoured at 3σ).

**
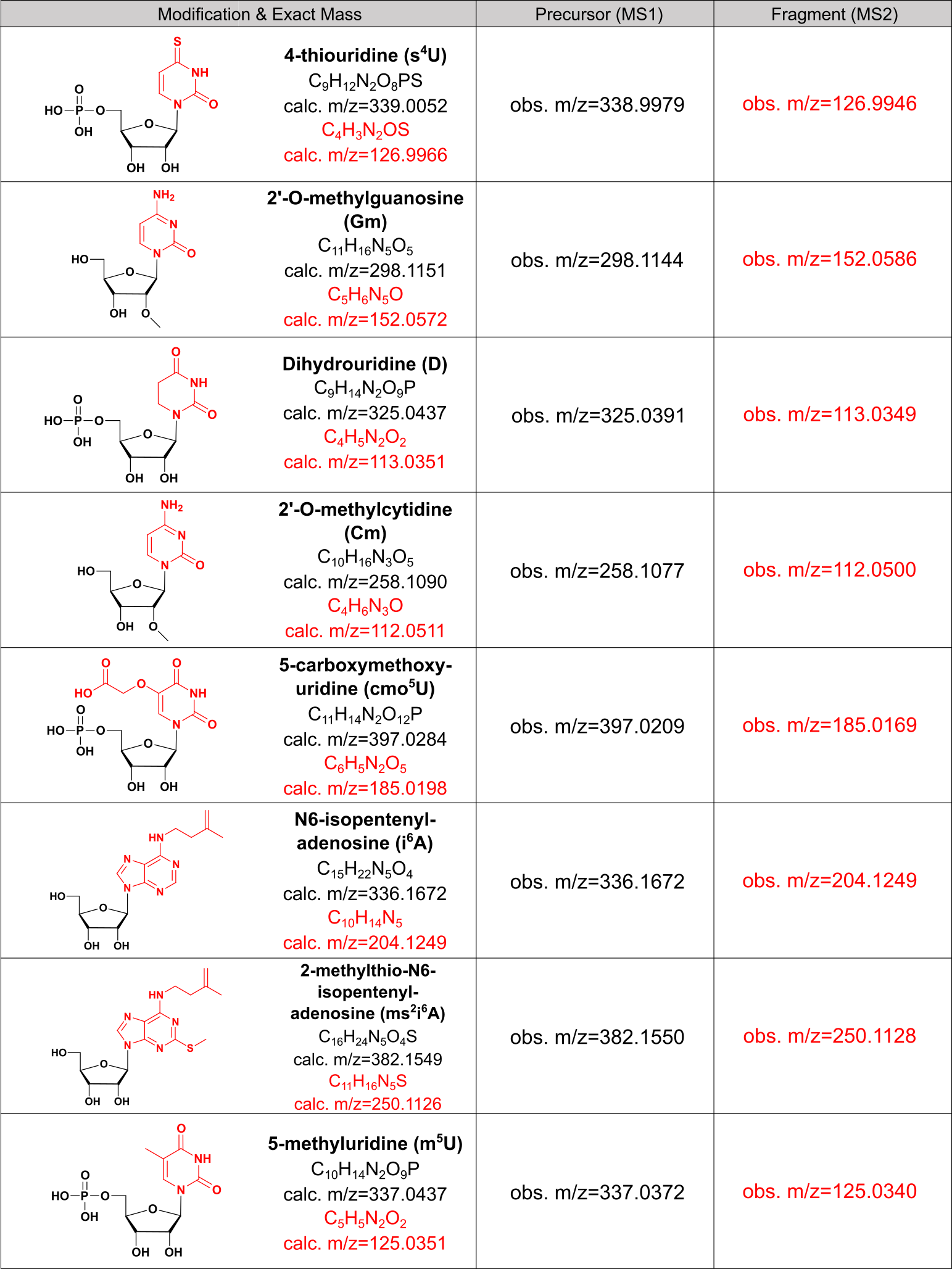
**

**Supplementary Figure 9.** **LC-MS/MS analyses of modifications present in tRNA^Ser1^(cmo^5^UGA)**. Chemical structures of precursor nucleosides/nucleotides are shown with the corresponding *m/z* (MS1) and the observed fragments in tandem mass spectrometry are labeled in red (MS2). Both N6-isopentenyladenosine (i^6^A) and 2-methylthio-N6-isopentenyladenosine (msi^6^A) were detected in the sample. calc. *m/z*, calculated *m/z*; obs. *m/z*. observed *m/z*.

**
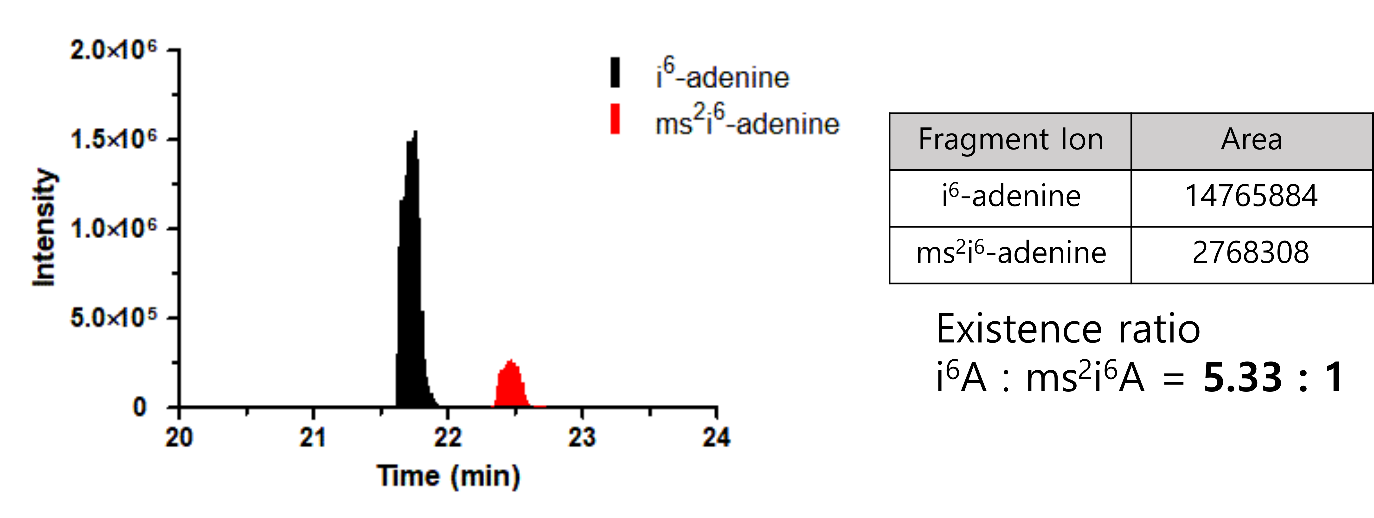
**

**Supplementary Figure 10. Multiple reaction monitoring (MRM) mode analysis of tRNA^Ser1^(cmo^5^UGA) containing i^6^A or ms^2^i^6^A**. Signature fragments of i^6^-adenine and ms^2^i^6^-adenine peaks were extracted from MRM mode of MS/MS spectra. Intensities were added up respectively to estimate the relative abundance.


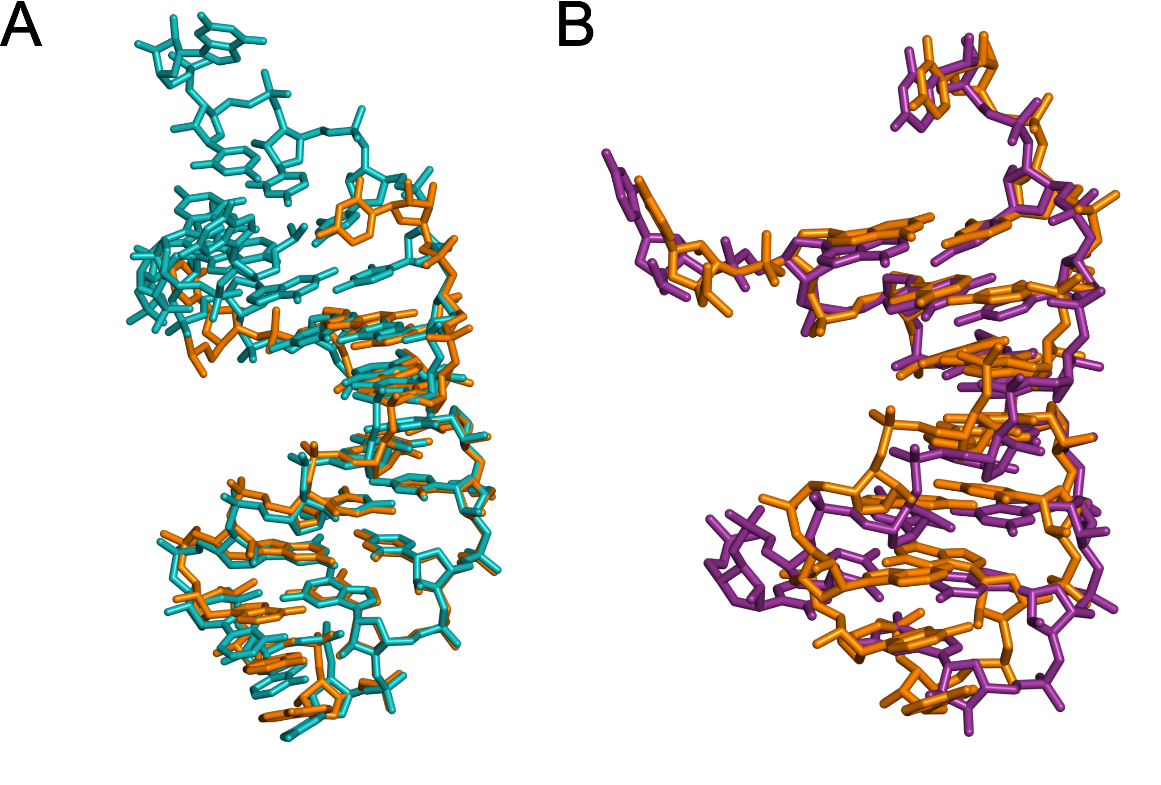


**Supplementary Figure 11. Structural comparison of variable arms.** The variable region of tRNA^Ser1^ (orange) is superimposed with that of A) *A. aeolicus* tRNA^Sec^ (PDB ID: 3W3S, teal) or B) *T. thermophilus* tRNA^Ser^ (PDB ID: 1SER, purple), which are complexed with seryl-tRNA synthetases (SerRS). The protein componets are not shown for clarity.
